# THE EFFECTS OF DRAINAGE TUBE ON PAIN AND FUNCTIONAL RECOVERY AFTER UNICOMPARTMENTAL KNEE ARTHROPLASTY

**DOI:** 10.1590/1413-785220243201e266853

**Published:** 2024-03-22

**Authors:** Ting Fu, Shuzhen Ren, Yu Nie

**Affiliations:** 1 Fuyang People’s Hospital, Fuyang, Anhui, China.; 2 Fuyang Second People’s Hospital, Fuyang, Anhui, China.

**Keywords:** Arthroplasty, Replacement, Knee, Drainage, Knee Joint, Morphine, Artroplastia do Joelho, Drenagem, Articulação do Joelho, Morfina

## Abstract

**Objective:**

The objective of this study was to evaluate the impact of drainage tube placement on postoperative pain, recovery, and opioid consumption within a 72-hour period following unicompartmental knee arthroplasty (UKA).

**Methods:**

Patients with medial knee osteoarthritis who underwent UKA from January 2019 to August 2020 were enrolled in the study and divided into two groups based on whether they received a drain postoperatively.

**Results:**

The drainage group had significantly lower VAS scores on day 1, day 2, and day 3, in addition to significantly smaller changes in the circumference of the knee joint within 3 days postoperatively (P <0.05). The ROM in the drainage group significantly increased at 3 days and 1 month post-surgery, with a statistically significant difference in morphine consumption between the two groups at 3 days (P<0.05). The incidence of postoperative nausea and vomiting (5 cases) and wound bleeding (1 case) was lower in the drainage group compared to the non-drainage group (P<0.05).

**Conclusions:**

The placement of a drainage tube in UKA may reduce the swelling of knee joint and pain, which not only reduces the use of Opioid but also facilitates early functional activities of the knee joint. ***Level of Evidence III; Retrospective Comparative Study.***

## RESUMO

**Objetivo:**

O objetivo deste estudo foi avaliar o impacto da implantação do tubo de drenagem na dor pós-operatória, na recuperação e no consumo de opioides em um período de 72 horas após a artroplastia unicompartimental do joelho (UKA).

**Métodos:**

Pacientes com osteoartrite medial do joelho submetidos à UKA de janeiro de 2019 a agosto de 2020 foram incluídos no estudo e divididos em dois grupos com base no fato de terem ou não recebido um dreno no pós-operatório.

**Resultados:**

O grupo de drenagem apresentou escores EVA significativamente menores no dia 1, no dia 2 e no dia 3, além de alterações significativamente menores na circunferência da articulação do joelho em 3 dias de pós-operatório (P <0,05). A ADM no grupo de drenagem aumentou significativamente em 3 dias e 1 mês após a cirurgia, com uma diferença estatisticamente significativa no consumo de morfina entre os dois grupos em 3 dias (P<0,05). A incidência de náuseas e vômitos no pós-operatório(5 casos) e sangramento da ferida (1 caso) foi menor no grupo de drenagem em comparação com o grupo sem drenagem (P<0,05).

**Conclusão:**

A utilização de tubo de drenagem na UKA pode reduzir o edema articular do joelho e a dor, reduzindo o uso de opioides e facilitando as atividades funcionais iniciais da articulação do joelho. ***Nível de Evidência III; Estudo Comparativo Retrospectivo.***

## INTRODUCTION

Unicompartmental knee arthroplasty (UKA) is one of the effective treatments for medial compartment osteoarthritis of the knee. When compared with total knee arthroplasty, UKA is minimally invasive that promotes faster postoperative functional recovery and shortens hospital length of stay.^1-4^ During UKA surgery, soft tissue incision and intraoperative osteotomy result in bleeding. Blood may then accumulate in the joint capsule and penetrate the soft tissue around the wound, leading to postoperative hematoma and swelling around the knee joint. Therefore, most surgeons would place a drainage tube in UKA surgery to prevent such complications.

In recent years, the application of multi-modal blood management in the perioperative period. Measures to reduce postoperative bleeding include the intraoperative controlled hypotension, tourniquet use, and the application of anti-fibrinolytic drugs.^5,6^ Due to the small size of the incision and relatively little bleeding in UKA, the need for placing a drainage tube has been debated.^7,8^ To date, few studies have examined the effects of drainage tubes after UKA on postoperative consumption of analgesics and early functional recovery. Therefore, this retrospective study investigated the impact of drainage tubes after UKA on postoperative pain, Opioid consumption during hospitalization, and early postoperative functional recovery in patients undergoing UKA for osteoarthritis of the medial compartment of the knee. Two main objectives drive this research. First, to investigate the effects of drainage tubes on pain and functional recovery after UKA. Second, to assess Opioid consumption over the first 3 days after surgery.

## METHODS

This was a single center, retrospective cohort study. The study was approved by the ethics committee of Fuyang People’s Hospital (2021-43), and written informed consent was obtained from patients and their family members. A total of 78 patients who had undergone medial single-compartment knee arthroplasty for osteoarthritis at our hospital from January 2019 to August 2020 were included. Other inclusion criteria were: (1) complete case and postoperative follow-up data; (2) osteoarthritis of the medial compartment of the knee, Primary UKA; (3) patients had normal coagulation profile, without hematological disorder and not consuming long-term oral hormones. Exclusion criteria included: (1) Incomplete case or follow-up data; (2) Inflammatory arthritis, which had an infection prior to surgery. Patients were assigned into two groups: Group A with a post-operative closed-suction drainage tube and Group B without a drainage tube. Preoperative basic and clinical demographics of patients are shown in Table 1. All patients were evaluated by the American Society of Anesthesiologists Classification Standards before surgery.

**Table 1:** Preoperative basic and clinical demographics between the two groups.

	Gender	Age (year of age)	BMI (kg/cm^2^)	HSS score	VAS score	ROM (^O^)
GroupA (n=40)	16/24	65.66±4.94	24.45±2.40	58.79±2.72	5.45±1.12	116.55±4.97
GroupB (n=38)	14/24	65.71+5.29	24.53±1.91	58.39±2.85	5.58±1.11	116.10±4.91
c^2^/F	0.082	0.094	2.286	O.001	0.26	0.17
P	0.774	0.871	0.796	0.628	0.605	0.519

### Surgical technique

Surgery was performed by the same treatment group and the same anesthetic team. All patients underwent combined spinal and epidural anesthesia. At 30 minutes before surgery, all patients received intravenous tranexamic acid 1.0 g. An oblique incision measuring approximately 6 cm in length was made on the medial side of the knee joint, and the parapatellar medial approach was taken. The skin, subcutaneous tissue, deep fascia and nodular capsules were incised in turn, the anterior and inner sides of the tibial plateau were exposed. The LINK unicondylar operation steps were followed, whereby the tibial osteotomy guide plate was placed for tibial osteotomy and the excised medial plateau bone block was completely removed. The thickness of the anterior and posterior margins was measured, and the meniscus remained after the removal. Then, the knee joint was straightened, the upper edge of the femoral side osteotomy was marked, the distal femur and posterior condyle cartilage were cut off in turn, and the tibial prosthesis trial model was installed. The flexion-extension gap and flexion-extension activity were measured to ensure no impact and the prosthesis looseness was appropriate before taking out the test model. After washing and drying, the cement was mixed, and the prosthesis was installed. The LINK®Sled unicondylar knee prosthesis was used in all patients. The activities of the knee joint flexion and extension and soft-tissue balance were tested after the solidification of the bone cement. The wound was then thoroughly rinsed, removed excess bone cement was removed, and hemostasis was ensured. In the drainage group, a negative pressure drainage tube was placed on the inner side of the knee. Finally, the wound was closed in layers with sutures.

### Postoperative management

All patients received anticoagulation and antibiotics postoperatively, with an intravenous drip of Parecoxib 40mg twice a day for 3 days. Opioid(Morphine hydrochloride)injection was administered according to the patient’s pain level. The drainage tube was removed 24 hours after surgery. Following the recovery of anesthesia, quadriceps active contraction exercise and straight leg elevation exercise were commenced, which encouraged early mobilization of patients after 1-2 days.

### Outcome measurements

The operative time (min), the postoperative pain level was assessed using the visual analog scale (VSA), and the VAS scores at day-1 to day-3 days after surgery in addition to the total consumption of morphine hydrochloride injection (mg) in the first 3 days were determined. The circumference of the knee joint and the knee joint mobility were measured on day-3 and day-6 following surgery. The number of patients requiring postoperative blood transfusion and incidences of nausea and vomiting were recorded. The knee function was assessed using the Hospital for Special Surgery (HSS) score at 1, 3, 6, and 12 months after surgery.

### Statistical analysis

All analyses were performed using IBM SPSS Statistics 24.0. Continuous variables were expressed as mean ± standard deviation with comparisons performed using the independent sample t-test. Categorical variables were presented as numbers (counts) with comparisons performed using the chi-square test. The correlation between the variables was analyzed by using the Pearson correlation. A P-value of <0.05 was considered statistically significant.

## RESULTS

There was no statistically significant difference between the two groups in gender, age, body mass index (BMI), preoperative knee HSS function score, VAS score, and the preoperative knee range of motion (ROM) (P>0.05) (Table 1).

Post-operative results are shown in Table 2. However, the drainage group had significantly lower VAS scores on day-1, day-2, and day-3, in addition to significantly smaller changes in the circumference of the knee joint within 3 days postoperatively (P <0.05). Also, the ROM of the knee joint at day-3 and 1 month was significantly higher in the drainage group but no difference was observed at 6 months following surgery when compared with the non-drainage group. The knee HSS function scores were significantly higher in the drainage group at 1 month and 3 months after surgery but no difference was observed at 6 months and 12 months after surgery.

**Table 2:** The operation time, total blood loss and postoperative outcomes between the two groups.

	Group A (n=40)	Group B (n=38)	F	P
Operation Time (min)	40±5.16	38±4.32	0.827	0.942
VAS score (postoperative)				
Day-1	3.67±0.67	4.23±0.88	0.031	0.004
Day-2	3.02±0.58	3.66±0.74	10.07	P < 0.001
Day-3	2.18±0.38	2.61±0.55	19.82	P < 0.001
Changes in the circumference of the knee joint on day-3 to baseline (cm)	2.95±0.30	3.45±0.29	0.075	P < 0.001
ROM(^O^)				
Day 3	100.42±5.22	91.53±3.43	4.439	P < 0.001
1st month	113.15±3.93	109±4.71	0.104	0.001
6th month	121.23±3.29	119.80±3.26	2.353	0.057
HSS score				
1st month	67.03±2.22	64.50±2.33	0.118	P < 0.001
3rd month	85.68±1.69	82.68±2.56	5.949	P < 0.001
6th month	89.80±1.78	89.13±1.53	0.012	0.08
12th month	93.18±1.71	92.89±1.64	0.011	0.463

**Figure 1:**
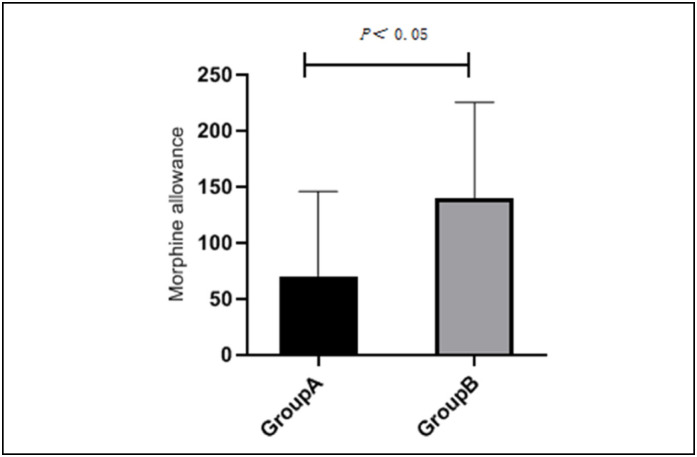
The amount of morphine hydrochloride between the two groups.

The incidence of postoperative nausea and vomiting was significantly higher in the nondrainage group, but no significant difference was observed between the groups in the requirement for blood transfusion, the incidence of wound infection, subcutaneous ecchymosis, or fat liquefaction. All the complications are shown in Table 3. The amount of morphine hydrochloride used within 3 days postoperatively was significantly different in either group (t=3.801, P<0.05) (Figure 1). Although the overall number of these adverse events was low, they were more apparent in the non-drainage group. All wound complications were resolved resolved without serious adverse consequences.

**Table 3:** Postoperative blood transfusion, wound complications and nausea and vomiting between the two groups.

	Requirement for blood transfusion	Nausea and vomiting	Wound infection	Ecchymosis	Fat liquefaction
Group A (n=40)	1	5	0	1	0
Group B (n=38)	0	15	1	3	2
c^2^	0.000	7.436	0.001	0.321	0.568
P	1.000	0.006	0.979	0.571	0.451

## DISCUSSION

Placement of a drainage tube in joint replacement surgery is common and widely practiced, which aimed to drain the blood in the joint cavity, reduce local tension, reduce inflammatory exudation and mitigate the risk of wound infection. The study by Kim et al^9^ has shown an increased risk of postoperative wound bleeding and potential wound infection if a drainage tube is not placed after knee arthroplasty. Li et al^10^ conducted a prospective randomized controlled study on 100 patients with total knee arthroplasty, showing that postoperative wound indwelling drainage tubes have no advantage in knee function recovery. However, patients without the drainage tube were more likely to experience blood stasis, tissue swelling, and blood extravasates. Anton et al^11^ conducted a randomized controlled study on 146 patients^12^ with total knee arthroplasty, and found no significant difference in blood loss, length of hospitalization, knee ROM, and KOOS score among patients with drainage tube placement. However, the value of a drainage tube after the UKA operation remains a controversy at present. In 2004, Confalonieri et al^13^ reported no clinical benefits of placing a drainage tube after UKA surgery but increased the cost of hospitalization. Nicola et al^14^ demonstrated placing a drainage tube did not provide substantial benefit in the management of blood loss after total knee arthroplasty or UKA. Furthermore, the study by Zhang et al^15^ has found that the blood loss and postoperative drainage volume of UKA were very low. Therefore, using drainage tubes in UKA did not confer any clinical advantages but increased the costs. On the other hand, Ares et al^12^ have conducted a study involving 234 patients undergoing knee arthroplasty and reported that the drainage tube could be used in patients with a large amount of intraoperative blood loss and a high risk of bleeding, and it should be removed at 24 hours after surgery. In our study, we found that patients in the drainage group had significantly better early knee ROM and the knee HSS function scores in the first and third months after surgery when compared with those in the non-drainage group.. These could be attributed to the placement of a drainage tube that reduces blood accumulation in the knee joint, leading to a reduction in the swelling around the knee joint. These would result in the low pressure in the capsule of the knee joint during training and the tension of soft tissue around the knee, contributing to reduced pain that is conducive to the patient’s early rehabilitation training.

In recent years, the promotion and practice of the rapid rehabilitation concept in perioperative management have successfully accelerated the postoperative functional recovery by reducing pain and postoperative complications, and consequently shorten the hospital length of stay and improve patient satisfaction.^16-18^ Early active functional exercise contributes to postoperative proprioception recovery that is vital for the daily activities of the knee joint. Hematoma in the joint capsule after UKA is one of the main causes of swelling around the knee. Joint swelling will then increase the tension around the knee joint and therefore, the patient will increase the muscle strength when completing the limb activity, resulting in increased pain at the knee joint, which then limits the active flexion and reduce the confidence of patients performing exercise during the early postoperative rehabilitation period. Pain is known as the "fifth-largest vital sign", and patients experiencing significant pain postoperatively may have increased fear and reluctant to participate in the rehabilitation training, leading to delayed recovery, increased length of hospital stay, and decreased patient satisfaction.^19,20^ Our analyses revealed that the pain level and usage of opioid usage were significantly lower in patients having a drainage tube than those without. Furthermore, a significantly higher incidence of postoperative nausea and vomiting was observed in the non-drainage group, which may result in patients’ poor oral intake, leading to hydro-electrolyte imbalance and increasing the need for intravenous fluid administration. Studies have shown that postoperative nausea and vomiting increase the length of hospital stay and the economic burden.^21^

The results show that the placement of a drainage tube after UKA reduces swelling of the knee joint swelling, lessens postoperative pain and opioid usage, facilitates early knee joint functional activities, and increases patient comfort and confidence in early rehabilitation training. Therefore, we recommend the placement of a drainage tube after UKA. However, there were limitations to this study. This was a retrospective study that included a relatively small sample size from a single center. Also, postoperative knee pain and nausea/vomiting might be multi-factorial and not solely due to the swelling of the knee joint or consumption of opioid-based analgesics, respectively. Therefore, a randomized control study is warranted to validate our findings.
